# 

**Published:** 1891-01-17

**Authors:** 


					OVrm
1^^ W II B Hi ?" BOVRlif"* "contains ^ the albumen and fibrinein the most perfect possible form, and to
T ? Mr SaS'SS55
P? any animal aliment at present known. C"T ^ SOLDtaTHROUGHOUT THE?.UNITED kin^uum.
S/wlPKrat/011 of meat, combining'in itself the albuminous'together with the extractive principles, nuou u preparauuu
would have to be preferred to the Extractum Oarnis, for it would contain all the nutritive constituents of meat." Again-" I have
it? a Si.aai^io fv.iU pre* ?-jrtv 'W/ \RT/ . paring the Extract of Meat, tlie Albuminous principles remain
they are ,HL?Igf *?st t? nutrition,^and this is certainly a great disadvantage."

				

